# Superficial Palmar Arch Aneurysm after Carpal Tunnel Decompression, a Rare Complication: A Case Report

**DOI:** 10.1155/2011/595120

**Published:** 2011-04-07

**Authors:** S. Gull, R. A. J. Spence, W. Loan

**Affiliations:** Belfast City Hospital, Lisburn Road, Belfast, BT9 7AB, UK

## Abstract

False aneurysms of the palmar arteries are rare. They are usually associated with traumatic injuries to the hand vasculature. We present a case of superficial palmar arch aneurysm (SPAA), complicating carpal tunnel decompression which presented as a pulsatile mass at the site of previous surgery. Initial diagnosis was made on clinical examination and confirmed on doppler ultrasound (US) and computed tomographic angiography (CTA). The feeding vessel of the aneurysm was subsequently occluded using coil embolization.

## 1. Case Presentation

**Figure 1:**
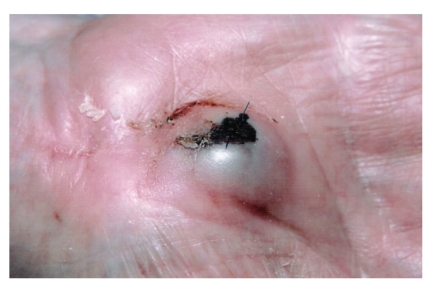
Pre-embolization picture of the swelling.

An 80-year-old male presented with a pulsatile mass (Figure 1) in his left hand 3 weeks following an open carpal tunnel decompression under general anaesthetic with tourniquet application. Tourniquet application time was less then 30 minutes, and it was released after wound closure and application of pressure dressing. Patient was discharged 24 hours after the surgery, and warfarin was restarted 72 hours after surgery with low molecular weight heparin cover.

His early postoperative course was complicated by surgical site abscess formation that was drained on postoperative day 7. There were no signs or symptoms of an embolic phenomenon secondary to infective endocarditis, since he was clinically well with no signs of generalised sepsis.

The patient had a past medical history of pulmonary embolism for which he was on warfarin along with previous ischaemic heart disease and minor asthma. 

**Figure 2:**
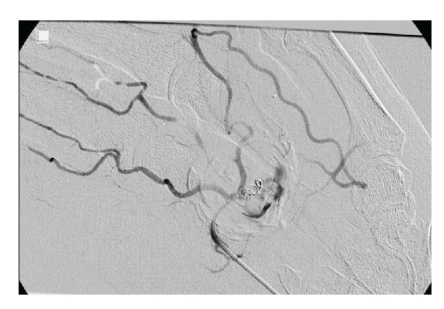
Coil in place in the pseudoaneurysm.

**Figure 3:**
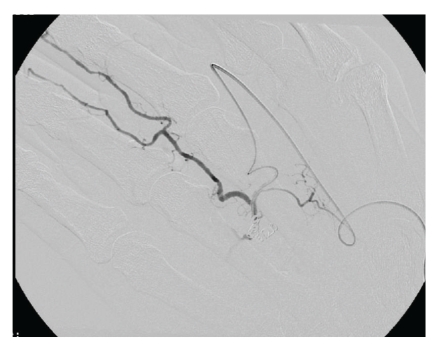
Postcoiling CTA confirming no flow in neck of pseudoaneurysm.

A Doppler ultrasound of his hand was performed which revealed arterial blood flow within the pulsatile swelling. CTA confirmed the lesion to be a pseudoaneurysm of the superficial palmer arch. Once the diagnosis was established, the patient's case was discussed in the vascular multidisciplinary meeting. He was provided with the options of open operative procedure under general anaesthesia versus endovascular technique, the latter was chosen by patient. Hence, coil embolization of both sides of the palmar arch (Figures 2 and 3) was carried out. A postprocedural US confirmed no flow into the aneurysm. His recovery was uneventful, and he was symptom free at 3-week followup. 

An initial diagnosis of mycotic aneurysm was made on the basis that patient initially presented with an abscess at the wound site on 7th postoperative day. This was drained in accident and emergency department under local anaesthetics. And the subsequent swelling appeared 2 weeks later.

 The patient did not have any history suggesting he suffered from “hypothenar hammer syndrome”, since he was a retired librarian, and there was no history of trauma to hand in the past.

## 2. Treatment

After informed consent for an endovascular procedure, the patients arm was supported in abduction, prepared, and draped, and the brachial artery was accessed under ultrasound guidance in antegrade direction. A 4 French sheath was inserted, and 5000 international units of heparin were given. 

Angiography, initially from the ulnar artery, confirmed the ultrasound and CTA findings, demonstrating a defect in the mid portion of the palmar arch, feeding the pseudo-aneurysm. A coaxial, 3 French (3F) microcatheter, and guide wire combination (Progreat, Terumo Japan) was used for angiography of the palmar arch. It proved impossible to pass the neck of the pseudo-aneurysm with a guide wire, and, therefore, the feeding artery was coiled (Tornado, Boston Scientific, USA) just to the ulnar side of the pseudo-aneurysm (Figure 2).

In order to fully isolate the pseudo-aneurysm, the radial artery was then selected, again using a 3F micro-catheter. The vessel was coiled very close to the pseudo-aneurysm from the radial approach, without affecting the digital branches (Figure 3).

A completion angiogram confirmed successful exclusion of the pseudo-aneurysm.

## 3. Discussion

A pseudo-aneurysm arises when a vessel wall is disrupted and surrounding tissues contain the resulting haematoma, whereas a true aneurysm involves dilation of the vessel including all layers of its wall [1]. 

 A pseudoaneurysm in the hand is rare, most resulting from injury to a digital artery [2–5]. 

The superficial palmar arch is a rare site for a pseudo-aneurysm, often leading to delayed or inaccurate diagnosis [6]. This condition is suspected when a pulsatile mass progressively increases in size [1]. Pseudoaneurysms are well-documented complications after surgery [7], arterial puncture, and trauma [7] and can develop after any procedure that causes partial disruption of vessel wall. A case of pseudo-aneurysm of the digital artery of the thumb associated with baseball injury has been reported [5]. 

Contrast enhanced computerized tomography angiography and high-resolution duplex colour Doppler are useful to confirm diagnosis. Angiography remains the most accurate way to delineate the anatomical detail of the pseudo-aneurysm and its collateral circulation [8]. However, angiography is controversial because of the potential complications, such as the risk of distal embolization [1]. 

Magnetic resonance angiogram is a useful and noninvasive imaging modality [9]. Surgical treatment comprises resection with arterial ligature [10] or arterial reconstruction [2, 3].

Restoration of the normal blood flow is preferable, especially in children [1]. All these investigations are equally helpful in making the diagnosis; in our case, US was performed followed by CT, but this can be changed according to local protocol of the unit.

Currently, very little has been published regarding SPAA. There have been 8 reported cases of SPAA in the English language literature, and none have been reported as a complication of a carpal tunnel release. In this case, the underlying aetiology may be resulting from surgical trauma or a complication of an infected hematoma. A criticism can be made that the decision to restart the patient's warfarin could have contributed to the complication in the early postoperative period.

 Endovascular embolization is an effective, minimally invasive technique for treating this rare complication. In our case, endovascular embolization was considered the first option after a discussion in multidisciplinary team meeting. Other options usually are open ligations, which involve complex microvascular surgery, open thrombectomy, and transversal arteriotomies; it was felt that an interventional radiological approach was most appropriate in this case.
